# Goniomitine: An Overview on the Chemistry of This Indole Alkaloid

**DOI:** 10.1155/2013/292396

**Published:** 2013-12-23

**Authors:** José C. F. Alves

**Affiliations:** Instituto de Pesquisas de Produtos Naturais Walter Mors, Centro de Ciências da Saúde, Bloco H, Universidade Federal do Rio de Janeiro, 21941-902 Rio de Janeiro, RJ, Brazil

## Abstract

This paper reports an overview on the chemistry of the indole alkaloid goniomitine focusing, mainly, on the methods of synthesis related to this natural product and analogs.

## 1. Introduction

**Figure 1:**
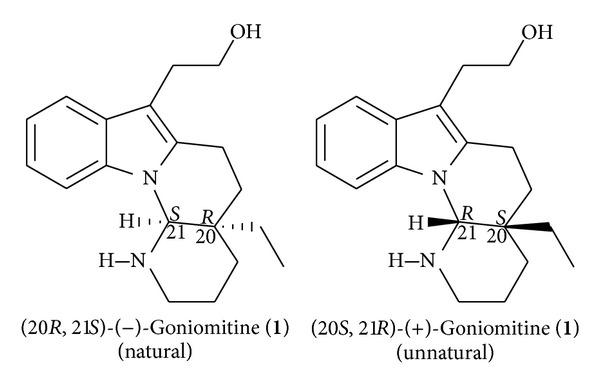
Natural (−)- and unnatural (+)-goniomitine (**1**).

The indole alkaloids belong to the class of natural substances displaying biological activities as well as a broad structural diversity. In view of these important properties, these products are target of study in the areas of isolation, identification, and synthesis [1–5]. Goniomitine (**1**) (Figure 1) is an indole alkaloid that was isolated and identified by Randriambola et al. [6] and Hashimoto and Husson [7]. The unique structure and biological activity of goniomitine have attracted the attention of several groups. This review describes the isolation, biogenesis hypothesis, chemical transformations, and syntheses of this alkaloid and analogs.

## 2. Isolation of Goniomitine

In the course of studies of the alkaloids of the genus *Gonioma*, Randriambola et al. [6] isolated, from the root bark of *Gonioma malagasy*, a crystalline compound named goniomitine with melting point of 150°C (ether-methanol), [*α*]_D_^20^ −80° (c 0.9 in CHCl_3_), and molecular formula C_19_H_26_N_2_O (HRMS, M^+∙^ 298.2080, calculated for 298.2045). The structure of goniomitine was initially proposed as indicated in Figure 1, with 20*S*, 21*R* configuration, based on its NMR spectra. Its absolute structure was deduced through the correlation with other alkaloids from *Aspidosperma* found in the same plant from where goniomitine had been isolated. The relative structure of goniomitine (**1**) was soon after confirmed by Takano et al. [8] through the total enantioselective synthesis of the natural form of this alkaloid. It could be evidenced that the absolute structure of the compound **1** is enantiomeric to the one that had been initially proposed for 20*S*, 21*R* configuration.

## 3. Biogenesis of Goniomitine

**Scheme 1:**
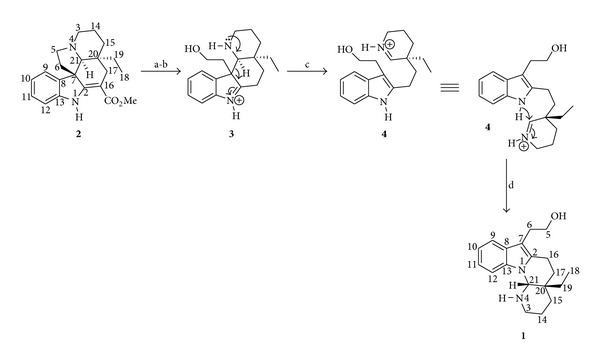
Biogenetic hypothesis of transformation of vincadifformine (**2**) into goniomitine (**1**): (a) oxidative fission of the C-5, N-4 bond; (b) decarboxylation; (c) retro-Mannich reaction; (d) nucleophilic attack of the indole nitrogen on the iminium moiety.

Randriambola et al. [6] proposed that goniomitine (**1**) may be derived from the *Aspidosperma* skeleton of vincadifformine (**2**) by the successive steps depicted in Scheme 1.

## 4. Chemical Transformations and Syntheses of Goniomitine and Analogs

### 4.1. Chemical Transformations of Goniomitine

**Scheme 2:**
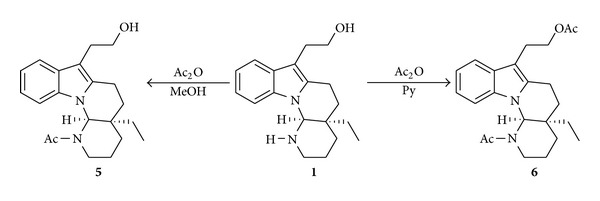
Chemical transformations of goniomitine (**1**) into the acetyl derivatives **5** and **6**.

For the occasion of the structural determination of goniomitine (**1**) [6], this compound was transformed into the *N*-acetyl derivative **5** upon treatment with Ac_2_O in MeOH and into the *N*,*O*-diacetyl derivative **6** upon treatment with Ac_2_O in pyridine (Scheme 2). The formation of the acetylated compounds **5** and **6** confirmed the presence of the groups OH and NH in the structure of **1**.

### 4.2. Synthesis of the Goniomitine Analog (+/−)-**12**

**Scheme 3:**
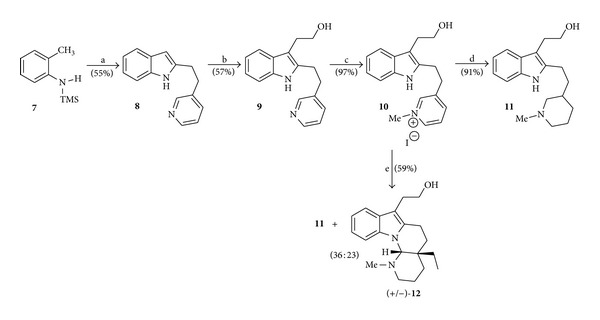
Reagents and conditions: (a) (i) *n*-BuLi (2.2 equiv), hexane (reflux, 6 h) and (ii) methyl 3-(3-pyridyl)propanoate, THF (−78 to 15°C); (b) MeMgI (10 equiv), ethylene oxide (10 equiv), Et_2_O (1 h), reflux (2 h); (c) MeI, CH_2_Cl_2_ (reflux, 2 h); (d) H_2_, PtO_2_, MeOH (3 h); (e) H_2_, PtO_2_, NaOMe, MeOH (3 h).

In order to ascertain unambiguously the unprecedented structure of the alkaloid goniomitine (**1**), Hashimoto and Husson [7] synthesized the goniomitine analog (+/−)-**12** by the sequence of reactions depicted in Scheme 3.

### 4.3. Total Synthesis of (−)-Goniomitine by Takano

**Scheme 4:**
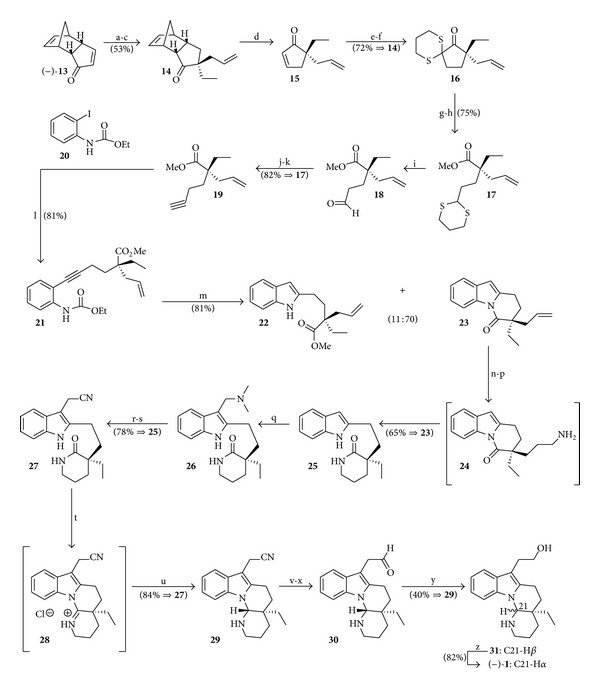
Reagents and conditions: (a) Zn (5.0 equiv), AcOH-EtOH (1 : 3), reflux (4 h); (b) EtI (2.0 equiv), *t*-BuOK (1.2 equiv), THF (−70 to −30°C, 15 min); (c) allyl bromide (2.0 equiv), *t*-BuOK (1.2 equiv), THF (−30°C, 5 min); (d) *o*-dichlorobenzene (reflux, 24 h); (e) LiAlH_4_ (1.0 equiv), CuI (0.5 equiv), HMPA-THF (1 : 4), −75°C (15 min); (f) propane-1,3-diyldithiotosylate (1.5 equiv), *t*-BuOK (3.0 equiv), *t*-BuOH-THF (1 : 4), 0°C; (g) KOH (5.0 equiv), *t*-BuOH (70°C, 12 h); (h) CH_2_N_2_, Et_2_O; (i) MeI (1.0 equiv), CaCO_3_ (5.0 equiv), 10% aq. MeCN (reflux, 1 h); (j) Ph_3_P (4.0 equiv), CBr_4_ (2.0 equiv), Et_3_N (3.0 equiv), CH_2_Cl_2_ (0°C, 5 min); (k) LDA (3.0 equiv), THF (−78°C, 10 min); (l) compound **20** (1.1 equiv), PdCl_2_(PPh_3_)_2_ (2%), CuI (5%), Et_3_N (reflux, 30 min); (m) NaOEt (10 equiv), Et_3_N (5%), EtOH (reflux, 3 h); (n) (i) dicyclohexylborane (1.5 equiv), THF (0°C, 30 min), (ii) 10% NaOH (1.0 equiv), 30% H_2_O_2_ (3.0 equiv), 0°C (30 min); (o) phthalimide (1.3 equiv), Ph_3_P (1.3 equiv), (*i*-PrO_2_CN)_2_ (1.3 equiv), THF (0°C, 10 min); (p) NH_2_NH_2_·H_2_O (4.0 equiv), EtOH (reflux, 2 h); (q) [Me_2_N=CH_2_]Cl (1.5 equiv), CH_2_Cl_2_ (r.t., 30 min); (r) MeI, MeOH (r.t., 10 min); (s) NaCN (1.3 equiv), DMF (100°C, 10 min); (t) POCl_3_ (6.0 equiv), toluene (reflux, 2 h); (u) NaBH_4_, MeOH, 0°C; (v) DIBAL (1.5 equiv), CH_2_Cl_2_ (−75°C, 10 min); (x) dil. H_2_SO_4_; (y) NaBH_4_; (z) 30% HCl-MeOH (1 : 10), reflux (30 min).

The first enantiocontrolled total synthesis of natural (−)-goniomitine (**1**) was published in 1991 by Takano et al. [8], who established the absolute stereochemistry of this alkaloid. This total synthesis, depicted in Scheme 4, starts with the chiral cyclopentadienone synthon (−)-**13**.

### 4.4. The First Biomimetic Approach to the Skeleton of Goniomitine from an *Aspidosperma* Alkaloid

**Scheme 5:**
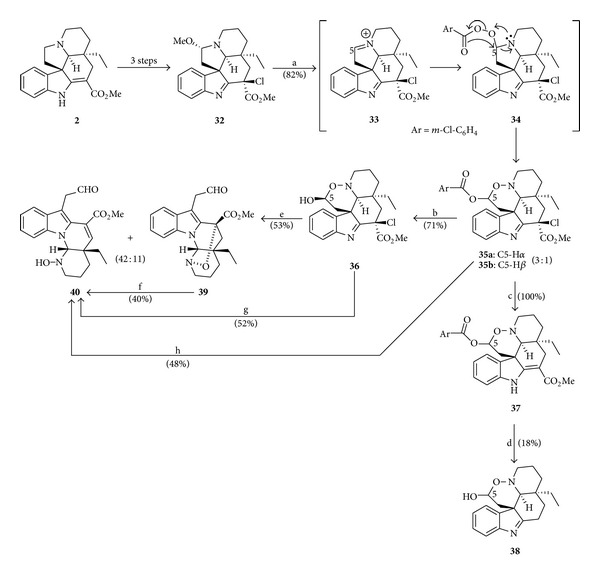
Reagents and conditions: (a) *m*-CPBA (1.1 equiv), CH_2_Cl_2_ (r.t., 3 h); (b) 0.2 mol L^−1^ NaOH-MeOH (r.t., 5 min); (c) NaI (3.0 equiv), AcOH (r.t., 1.5 h); (d) 11 mol L^−1^ HCl (105°C, 10 min); (e) TFA (16 equiv), CH_2_Cl_2_ (r.t., 20 min); (f) TFA (r.t., 4 h); (g) TFA (16 equiv), CH_2_Cl_2_ (r.t., 15 h); (h) TFA (12.5 equiv), CH_2_Cl_2_ (r.t., 45 h).

**Scheme 6:**
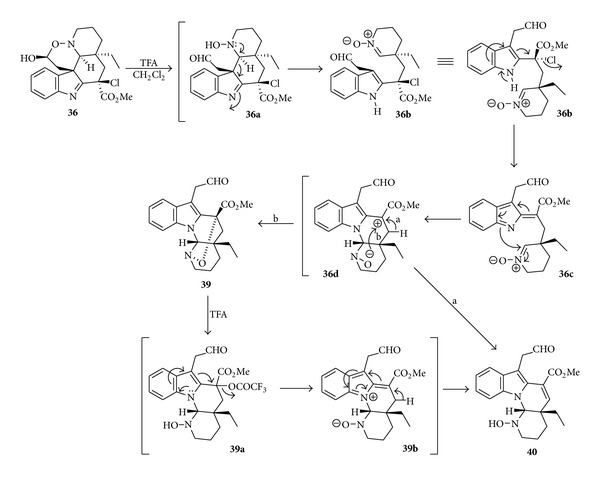


The results from the study of biomimetic transformation of an *Aspidosperma *alkaloid (**2**) into the substances **39**-**40**, with the skeleton of goniomitine (**1**), were published in 1995 by Lewin et al. [9]. The sequences of reactions for the discovery of a new biomimetic *in vitro* rearrangement are depicted in Scheme 5.  Scheme 6 displays the proposed mechanism [9] for the transformation of compound **36** into the alkaloids **39** and **40**.

### 4.5. Semisynthesis of (+)-(16*S*,20*S*,21*R*)-16-Hydroxymethyl-goniomitine from (−)-Vincadifformine

**Scheme 7:**
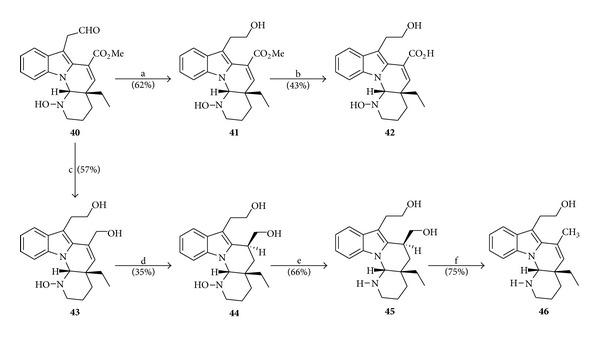
Reagents and conditions: (a) NaBH_3_CN, AcOH (r.t., 1.5 h); (b) NaOH-MeOH (120°C, 1 h); (c) LiAlH_4_ (*excess*), THF (reflux, 3 h); (d) H_2_ (1 atm), 10% Pd-C, MeOH (r.t., 5 h); (e) TiCl_3_-H_2_O, MeOH (r.t., 20 h); (f) 30% HCl-MeOH (120°C, 1.5 h).

In continuation to the studies of chemical transformations of vincadifformine (**2**) into alkaloids analogs to goniomitine (**1**), Lewin and Schaeffer [10] published in 1995 the semisynthesis of (+)-16-hydroxymethyl-goniomitine (**45**). This alkaloid was obtained as a result of the attempts to synthesize (+)-goniomitine (**1**) from the compound **40**, previously obtained from (−)-vincadifformine (**2**) (Scheme 5) [9]. In Scheme 7 are depicted the sequences of reactions that led to the synthesis of compound **45** as well as other alkaloids with tetracyclic skeleton of goniomitine (**1**).

### 4.6. Synthesis of the Goniomitine Analogs **52**–**55** by Cycloaddition Reactions

**Scheme 8:**
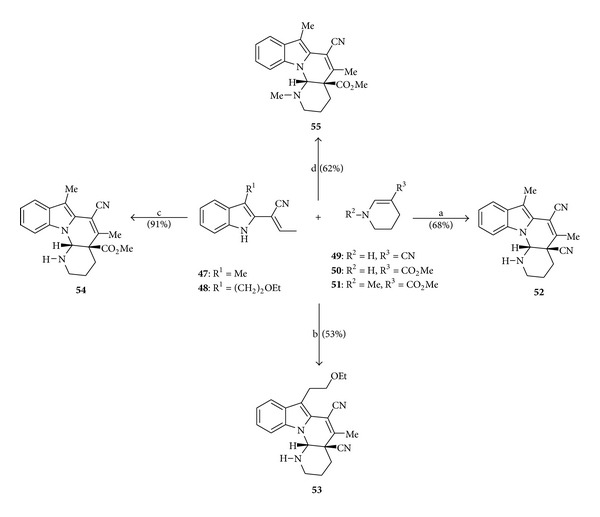
Reagents and conditions: (a) vinylindole **47** (1.0 equiv), enamine **49** (2.37 equiv), CH_3_CN, LiClO_4_ (0.1 mol L^−1^), electrolysis (480 mV *versus* Ag/AgNO_3_, current (20 to 2 mA), 200 min); (b) vinylindole **48** (1.0 equiv), enamine **49** (6.17 equiv), CH_3_CN, LiClO_4_ (0.1 mol L^−1^), electrolysis (480 mV *versus* Ag/AgNO_3_, current (20 to 2 mA), 200 min); (c) vinylindole **47** (1.0 equiv), enamine **50** (1.4 equiv), CH_3_CN, LiClO_4_ (0.1 mol L^−1^), electrolysis (480 mV *versus* Ag/AgNO_3_, current (20 to 2 mA), 40 min); (d) vinylindole **47** (1.0 equiv), enamine **51** (2.1 equiv), CH_3_CN, LiClO_4_ (0.1 mol L^−1^), electrolysis (480 mV *versus* Ag/AgNO_3_, current (20 to 2 mA), 200 min).

In the year 1996, Gürtler et al. [11] published the synthesis of the goniomitine analogs **52**–**55** by [4 + 2] cycloaddition reactions between 2-vinylindoles and substituted cyclic enamines, via anodic oxidation (Scheme 8).

### 4.7. Proposal of Synthesis of Goniomitine by Alves

In the year 2000, Alves [12] presented his qualification exam of doctorate about a plan of synthesis of the indole alkaloid goniomitine (**1**). The convergent strategies and synthetic routes for the synthesis of this alkaloid, idealized on that occasion, are described in the supplementary material of this review, available online at http://dx.doi.org/10.1155/2013/292396.

### 4.8. Syntheses of Cytotoxic Bisindole Alkaloids

**Scheme 9:**
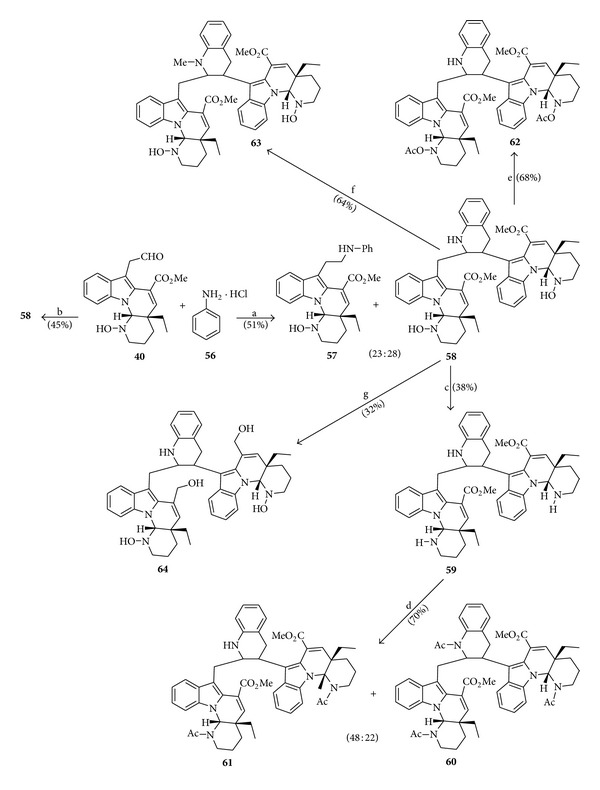
Reagents and conditions: (a) compound **56** (5.0 equiv), NaBH_3_CN (*immediate addition*), MeOH (r.t., 16 h); (b) compound **56** (5.0 equiv), NaBH_3_CN (*delayed addition*, 20 min), MeOH (r.t., 16 h); (c) TiCl_3_-H_2_O (6.0 equiv), MeOH (r.t., 20 h); (d) Ac_2_O, Py (r.t., 48 h); (e) Ac_2_O, Py (r.t., 3 h); (f) CH_2_O, NaBH_3_CN, AcOH (r.t., 2 h); (g) LiAlH_4_, THF (reflux, 3 h).

In the year 2000, Lewin et al. [13] published an article about a slight modification of the Borch reductive amination method (delayed addition of NaBH_3_CN) [14, 15], applied to compound **40**, analog of the natural alkaloid goniomitine (**1**). As a result of this reaction, a series of new cytotoxic bisindole alkaloids was prepared, as depicted in Scheme 9.

In continuation to the studies of synthesis of cytotoxic bisindole alkaloids, Raoul et al. [16] published, in the year 2001, an article with a novel series of these alkaloids prepared by reductive amination of the compound **40** with various anilines, using the modified Borch amination conditions described in Scheme 9 (delayed addition (20 min) of NaBH_3_CN) [15]. The influence of substitution of the starting aniline on the reaction and on cytotoxicity of produced dimers is discussed in the paper.

### 4.9. Total Synthesis of (+/−)-Goniomitine by Pagenkopf

**Scheme 10:**
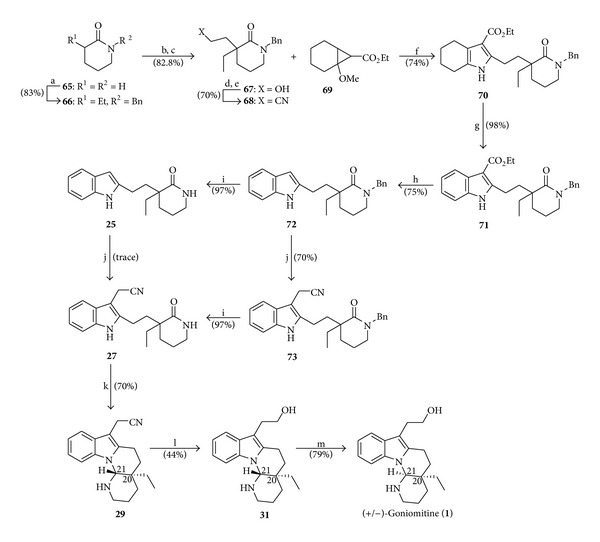
Reagents and conditions: (a) (i) *n*-BuLi (2.0 equiv), THF (−78°C), (ii) EtI (1.0 equiv), −78°C (1 h), (iii) BnBr (1.0 equiv), r.t. (*overnight*); (b) (i) LDA (1.0 equiv), THF (−78°C, 15 min), (ii) BrCH_2_CH_2_OTHP (1.1 equiv), r.t. (*overnight*); (c) TsOH (0.1 equiv), MeOH (*ice-brine bath*, 4 h); (d) Et_3_N (2.1 equiv), MsCl (1.0 equiv), CH_2_Cl_2_ (0°C to r.t., 3 h); (e) NaCN (2.0 equiv), MeCN, 120°C (**μ**w, 8 h, 900 rpm stirring); (f) Nitrile **68** (1.0 equiv), cyclopropane **69** (2.9 equiv), TMSOTf (1.0 equiv), EtNO_2_ (−30°C, 24 h); (g) 5% Pd-C (0.03 equiv), mesitylene (reflux, 24 h); (h) NaOH (10 equiv), EtOH-H_2_O (1 : 1), 150°C (**μ**w, 3 h, 900 rpm stirring); (i) Na (5.0 equiv), *liq.* NH_3_ (0.042 mol L^−1^), THF (−78°C, 10 min); (j) (i) [Me_2_N=CH_2_]Cl (1.5 equiv), CH_2_Cl_2_ (r.t., 15 min), (ii) MeI (40 equiv), MeOH (r.t., 10 min), (iii) NaCN (1.3 equiv), DMF (100°C, 10 min); (k) (i) POCl_3_ (6.0 equiv), toluene (reflux, 2 h), (ii) NaBH_4_ (2.0 equiv), MeOH (0°C, 30 min); (l) (i) DIBAL (1.5 equiv), CH_2_Cl_2_ (−78°C, 10 min), (ii) 0.75 mol L^−1^ H_2_SO_4_, (iii) NaBH_4_ (2.2 equiv), EtOH (0°C, 30 min); (m) TsOH (*cat*.), Et_3_N-MeOH (3 : 5, v/v), reflux (30 min).

In the year 2008, Morales and Pagenkopf [17] published the total synthesis of racemic (+/−)-goniomitine (**1**), accomplished in 17 linear steps with 5.2% overall yield starting from commercially available *δ*-valerolactam (**65**). Their synthetic approach includes the application of a formal [3+2] cycloaddition between the highly functionalized nitrile **68** and the activated cyclopropane **69** to prepare the indole nucleus (Scheme 10).

### 4.10. Total Synthesis of (+/−)-Goniomitine by Waser

**Scheme 11:**
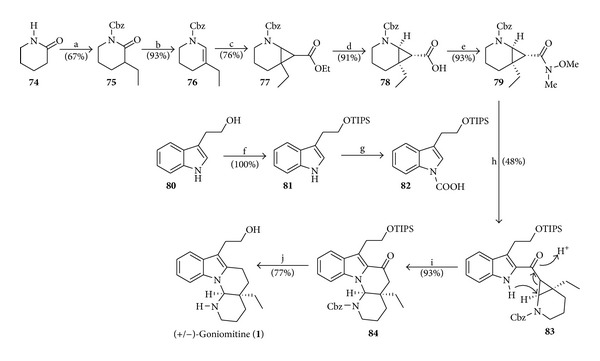
Reagents and conditions: (a) (i) *n*-BuLi (2.2 equiv), THF (0°C, 30 min), (ii) EtI (1.5 equiv), 0°C (20 min), (iii) benzyl chloroformate (1.05 equiv), 0°C (20 min); (b) (i) NaBH_4_ (1.05 equiv), MeOH (0°C, 15 min), (ii) conc. H_2_SO_4_, Et_2_O (r.t., 1 h); (c) N_2_CH_2_COOEt (4.0 equiv), (CuOTf)_2_·C_7_H_8_ (0.02 equiv), CH_2_Cl_2_ (18 h); (d) (i) BF_3_·OEt_2_ (0.15 equiv), CH_2_Cl_2_ (−20 to 0°C), (ii) NaOH (9.0 equiv), H_2_O-THF-EtOH (1 : 1 : 3), 0°C to 60°C (2 h); (e) (i) DMTMM (1.5 equiv), THF (r.t., 60 min), (ii) MeNHOMe.HCl (1.0 equiv), NMM (2.0 equiv), r.t. (36 h); (f) TIPSCl (1.05 equiv), imidazole (2.1 equiv), DMF (r.t., 1 h); (g) (i) *n*-BuLi (1.2 equiv), Et_2_O (0°C then reflux, 2 h), (ii) CO_2_ (0°C, 30 min), (iii) H_3_O^+^ (pH 2); (h) (i) *t*-BuLi (3.0 equiv), compound **82** (1.5 equiv), TMEDA (2.0 equiv), THF (−78°C, 3 h), (ii) amide **79** (1.0 equiv), THF (0°C, 20 min); (i) TsOH (0.2 equiv), CH_2_Cl_2_ (r.t., 10 min); (j) (i) NaBH_4_, MeOH (0°C to r.t., 3 h), (ii) Ac_2_O, Py (r.t., *overnight*), (iii) H_2_, Pd-C (0.1 equiv), EtOH, (iv) TBAF (4.4 equiv), THF (r.t., 30 min).

De Simone et al. [18] published the synthesis of racemic goniomitine (**1**) with the first study of its bioactivity, revealing significant cytotoxicity against several cancer cell lines [18, 19]. The strategy of this synthesis is based on cyclization of aminocyclopropanes [20], applied to cyclopropyl ketone **83** to lead to compound **84** with tetracyclic skeleton of goniomitine (Scheme 11).

### 4.11. Total Syntheses of (+/−)-, (−)-, and (+)-Goniomitine by Mukay

**Scheme 12:**
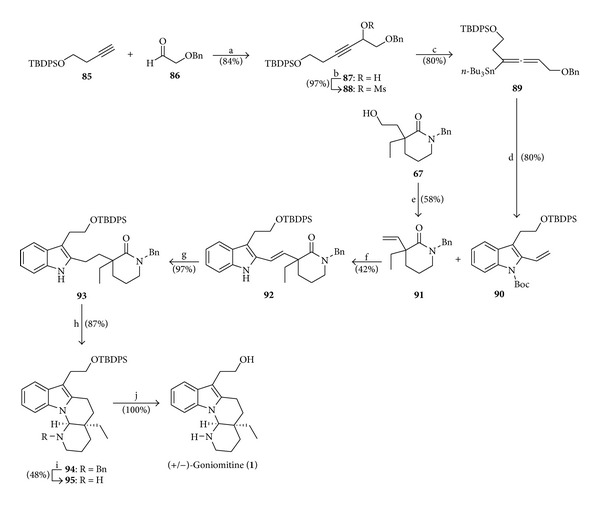
Reagents and conditions: (a) (i) compound **85** (1.3 equiv), *n*-BuLi (1.2 equiv), THF (−78°C, 1.5 h), (ii) compound **86** (1.0 equiv), THF (r.t., 22 h); (b) MsCl (1.6 equiv), Et_3_N (2.0 equiv), CH_2_Cl_2_ (0°C to r.t., 20 min); (c) (i) LDA (2.4 equiv), *n*-Bu_3_SnH (2.4 equiv), THF (−78°C, 1 h), (ii) CuBr·SMe_2_ (2.7 equiv), −78°C (40 min), (iii) mesylate **88** (1.0 equiv), THF (−78°C, 1 h); (d) (i) 2-I-PhNHBoc (1.28 equiv), compound **89** (1.0 equiv), TBAC (3.29 equiv), TFP (0.25 equiv), Pd_2_(dba)_3_ (0.03 equiv), CuI (0.11 equiv), DMF (r.t., 2 h); (e) (i) *o*-NO_2_PhSeCN (1.54 equiv), *n*-Bu_3_P (1.55 equiv), THF (r.t., 5 h), (ii) 30% aq. H_2_O_2_ (1.48 mol L^−1^), THF (0°C (20 min), r.t. (17 h)); (f) compound **90** (1.0 equiv), lactam **91** (9.44 equiv), Hoveyda-Grubbs-II cat. (0.3 equiv), neat (140°C, 3 h); (g) H_2_, 5% Pd-C (0.1 equiv), AcOEt (r.t., 23 h); (h) DIBAL (3.4 equiv), THF (−78°C to r.t.); (i) H_2_, 20% Pd (OH)_2_, AcOH-EtOH (5 : 2), r.t. (2 h); (j) TBAF (3.3 equiv), THF (r.t., 14 h).

In the year 2011, Mizutani et al. [21] published the syntheses of both racemic and optically active goniomitine, whose principal steps are the preparation of the indole skeleton by their own developed procedure [22] and alkene cross-metathesis. The synthesis of racemic (+/−)-goniomitine (**1**) was performed, as a preliminary study, by the sequence of reactions depicted in Scheme 12.

**Scheme 13:**
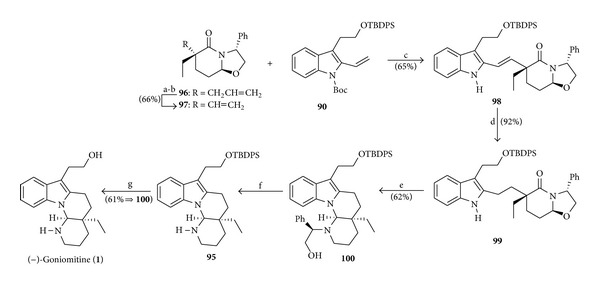
Reagents and conditions: (a) (i) O_3_, MeOH (−78°C, 15 min) and (ii) NaBH_4_ (1.5 equiv), −78°C to r.t. (2 h); (b) (i) *o*-NO_2_PhSeCN (3.9 equiv), *n*-Bu_3_P (6.0 equiv), THF (r.t., 3 h) and (ii) 30% aq. H_2_O_2_, THF (0°C to r.t., 9 h); (c) indole **90** (1.0 equiv), lactam **97** (3.5 equiv), Hoveyda-Grubbs-II cat. (0.31 equiv), xylene (140°C, 3 h); (d) H_2_, 5% Pd-C (0.1 equiv), AcOEt (r.t., 27 h); (e) (i) NaH (17.8 equiv), Et_2_O (0°C, 30 min) and (ii) DIBAL (1.07 equiv), 0°C to r.t. (10 min), repeat three-times; (f) H_2_, 20% Pd (OH)_2_, *n*-PrOH/1,4-dioxane (1 : 1), r.t. (11 h); (g) TBAF (3.3 equiv), THF (r.t., 14 h).

The convergent total synthesis of the natural (−)-goniomitine (**1**) [21] was completed by the sequence of reactions depicted in Scheme 13.

Using the synthetic route described in Scheme 13, but starting from the enantiomer of the lactam **97** (*ent-* **97**) Mizutani et al. [21] synthesized the unnatural (+)-goniomitine (*ent*-**1**). With the racemic, natural, and unnatural goniomitine in hand, the authors [21] executed the preliminary bioactive assays, which revealed that natural (−)-goniomitine has stronger antiproliferative activity in Mock and MDCK/MDR1 cells than its enantiomer.

### 4.12. Total Synthesis of (+/−)-Goniomitine by Bach

**Scheme 14:**
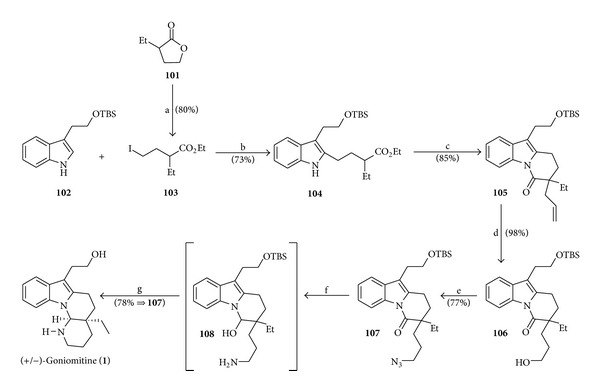
Reagents and conditions: (a) (i) NaI (1.5 equiv), TMSCl (1.5 equiv), MeCN (r.t., 30 min), (ii) lactone **101** (1.0 equiv), MeCN (r.t., 16 h), (iii) TMSCl (0.5 equiv), EtOH (r.t., 71 h); (b) compound **102** (1.0 equiv), norbornene (2.01 equiv), K_2_CO_3_ (4.01 equiv), iodide **103** (4.01 equiv), PdCl_2_ (0.1 equiv), DMF-DMSO (9 : 1), H_2_O (0.5 mol L^−1^), air (60°C, 26 h); (c) (i) indole **104** (1.0 equiv), LiHMDS (3.0 equiv), THF (−78°C to r.t.), (ii) CH_2_=CHCH_2_Br (3.0 equiv) (−78°C (40 min), r.t. (30 min)); (d) (i) lactam **105** (1.0 equiv), 9-BBN (1.39 equiv), (0°C (15 min), r.t. (1 h)), (ii) aq. NaOH (1 mol L^−1^), 35% aq. H_2_O_2_ (0.18 mol L^−1^), 0°C (30 min); (e) alcohol **106** (1.0 equiv), PPh_3_ (2.08 equiv), DPPA (2.94 equiv), DIAD (2.8 equiv), 0°C to r.t. (3.5 h); (f) azide **107** (1.0 equiv), LiAlH_4_ (4.01 equiv), THF (0°C to r.t., 2 h); (g) AcOH-THF-H_2_O (3 : 1 : 1, v/v), 40°C (24 h).

In the year 2012, Jiao et al. [23] published the total synthesis of racemic goniomitine (**1**), using the strategy of C-2 alkylation of indoles catalyzed by palladium via a norbornene-mediated C–H activation [24]. The steps for the synthesis of (+/−)-goniomitine (**1**), by this strategy, are depicted in Scheme 14.

### 4.13. Synthesis of (+)- and (−)-Goniomitine by Lewin

**Scheme 15:**
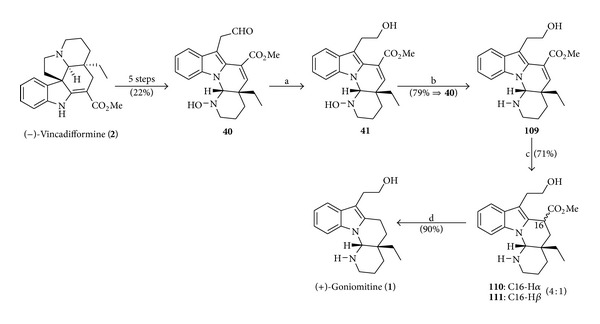
Reagents and conditions: (a) compound **40** (1.0 equiv), NaBH_3_CN (5.73 equiv), AcOH (r.t., 2.5 h); (b) TiCl_3_ (3.1 equiv), MeOH (r.t., 20 h); (c) compound **109 **(1.0 equiv), HCO_2_NH_4_ (5.71 equiv), 10% Pd-C (0.33 equiv), MeOH (reflux, 45 min); (d) 4 mol L^−1^ HCl (100°C, 1 h).

In the year 2013, Lewin et al. [25] have published the first biomimetic semisynthesis of goniomitine (**1**), in nine steps with 11% overall yield, starting from vincadifformine (**2**). Natural (−)- and unnatural (+)-goniomitine were prepared from (+)- and (−)-vincadifformine, respectively. The steps for the synthesis of unnatural (+)-goniomitine (**1**) are depicted in Scheme 15.

Lewin et al. [25] have synthesized the natural (−)-goniomitine (**1**), starting from (+)-vincadifformine (*ent*-**2**), using the same conditions described in Scheme 15. The evaluation of the antiproliferative effect of (+)- and (−)-goniomitine (**1**), undertaken on five human cancer cell lines, has demonstrated that unnatural (+)-goniomitine is more potent than its enantiomer (−)-goniomitine [25], in opposition to Mizutani et al.'s results on a canine kidney cell line (MDCK II) [21].

### 4.14. Synthesis of (+/−)-Goniomitine by Zhu

**Scheme 16:**
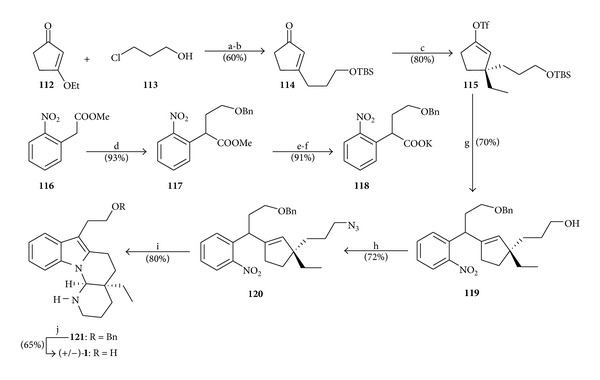
Reagents and conditions: (a) (i) compound **113** (2.0 equiv), CH_3_MgCl (2.0 equiv), THF (−78°C to r.t.), (ii) Mg (2.2 equiv), reflux (3 h), (iii) compound **112** (1.0 equiv), THF (reflux, 2 h), (iv) 2 mol L^−1^ HCl (0°C, 3 h); (b) TBSCl (1.1 equiv), imidazole (1.5 equiv), DMF (r.t, 3 h); (c) (i) CuBr·Me_2_S (2.0 equiv), EtMgBr (4.0 equiv), THF (−78 to −40°C, 40 min), (ii) compound **114** (1.0 equiv), THF (−40°C, 3 h), (iii) Comins' reagent (2.0 equiv), THF (r.t., 24 h); (d) compound **116** (1.0 equiv), ICH_2_CH_2_OBn (1.2 equiv), Cs_2_CO_3_ (1.3 equiv), DMF (60°C, *overnight*); (e) compound **117** (1.0 equiv), 10% aq. KOH, MeOH-THF (5 : 1), r.t. (5-6 h); (f) *t*-BuOK (1.0 equiv), EtOH (r.t., 1 h); (g) (i) compound **118** (1.2 equiv), [PdCl(allyl)]_2_ (5 mol%), X-Phos (15 mol%), triflate **115** (1.0 equiv), diglyme (100°C, 2 h), (ii) TBAF (4.0 equiv), THF (r.t., 4 h); (h) compound **119** (1.0 equiv), Ph_3_P (2.1 equiv), DPPA (2.9 equiv), DIAD (2.8 equiv), THF (0°C to r.t., 3.5 h); (i) (i) compound **120** (1.0 equiv), NaHCO_3_ (5.0 equiv), MeOH, O_3_ (−78°C, 20–30 seg), (ii) Me_2_S (50 equiv), −78°C to r.t. (24 h), (iii) Zn (70 equiv), CaCl_2_ (20 equiv), MeOH (reflux, 2 h); (j) compound **121** (1.0 equiv), sodium naphthalenide (6.0 equiv), THF (−20°C, 15 min).

In the year 2013, Xu et al. [26] have published a seven-step total synthesis of (+/−)-goniomitine (**1**) through two key steps: (i) a novel palladium-catalyzed decarboxylative coupling reaction between the potassium nitrophenyl acetate **118** and the vinyl triflate **115** for a rapid production of the functionalized cyclopentene **119**; (ii) a late-stage construction of the whole tetracyclic scaffold of goniomitine (**1**) from the functionalized cyclopentene **120** by a one-pot integrated oxidation/reduction/cyclization (IORC) sequence (Scheme 16).

## 5. Conclusions

In summary, it may be concluded that this brief survey on the chemistry of goniomitine has covered the literature relative to this alkaloid and analogs from 1987 to the first semester of the year 2013. Taking into account the results published in this period, a considerable progress on the synthesis of this alkaloid has been verified in the last years (2008–2013) with the publications of five racemic and two enantiomeric syntheses. It is also important to emphasize the recent pioneering works on the bioactive assays performed with the racemic mixtures as well as both enantiomers of goniomitine. In spite of these progresses, the development of new efficient enantioselective synthetic strategies for this indole alkaloid, with low operational costs, is still a target to be reached.

## Supplementary Material

This supplementary material displays the proposals of synthesis of the indole alkaloids (+/-)-goniomitine, the natural (-)-goniomitine, and the unnatural (+)-goniomitine. The idealized strategies and synthetic routes for the preparation of these alkaloids and stereisomers are described.

## Abbreviations

Ac: Acetyl
9-BBN: 9-Borabicyclo[3.3.1] nonane
Boc: *tert*-Butoxycarbonyl
Bn: Benzyl
*n*-Bu: *n*-Butyl
*t*-Bu: *tert*-Butyl
Bz: Benzoyl
DIAD: Diisopropyl azodicarboxylate
DIBAL: Diisobutylaluminum hydride
DMF: *N*,*N*-Dimethylformamide
DMSO: Dimethyl sulfoxide
DMTMM: 2,4-Dimethoxy-6-(4-methylmorpholin-4-ium-4-yl) chloride
DPPA: Diphenylphosphoryl azide
Et: Ethyl
HMPA: Hexamethylphosphoramide
LDA: Lithium diisopropylamide
LiHMDS: Lithium bis(trimethylsilyl)amide
*m*-CPBA: *meta*-Chloroperbenzoic acid
Me: Methyl
Ms: Mesyl (methanesulfonyl)
NMM: *N*-methylmorpholine
Pd_2_(dba)_3_: Tris(dibenzylideneacetone)dipalladium(0)
*i*-Pr: *iso*-Propyl
*n*-Pr: *n*-Propyl
Ph: Phenyl
Py: Pyridine
TBAC: Tetrabutylammonium chloride
TBAF: Tetrabutylammonium fluoride
TBDPS: *tert*-Butyldiphenylsilyl
TBS: *tert*-Butyldimethylsilyl
Tf: Trifluoromethanesulfonyl
TFA: Trifluoroacetic acid
TFP: Tetrafluorophenyl
THF: Tetrahydrofuran
THP: Tetrahydropyranyl
TIPSCl: Triisopropylsilyl chloride (chlorotriisopropylsilane)
TMEDA: *N*, *N*, *N*′, *N*′-Tetramethylethylenediamine
TMSCl: Trimethylsilylchloride
TMSOTf: Trimethylsilyl trifluoromethanesulfonate
Ts: Tosyl (*p*-toluenesulfonyl)
X-Phos: 2-Dicyclohexylphosphino-2′,4′,6′-triisopropylbiphenyl.
