# Supplementary material for: Goniomitine: An Overview on the Chemistry of This Indole Alkaloid
Source: ISRN Org Chem. 2013 Dec 23;2013:292396. doi: 10.1155/2013/292396 (PMC3884615; doi:10.1155/2013/292396)
Supplement: Supplementary file 1 — This supplementary material displays the proposals of synthesis of the indole alkaloids (+/-)-goniomitine, the natural (-)-goniomitine, and the unnatural (+)-goniomitine. The idealized strategies and synthetic routes for the preparation of these alkaloids and stereisomers are described. [file 292396.f1.pdf]

Supplementary Information

Goniomitine: An Overview on the Chemistry of This Indole Alkaloid

José C. F. Alves

Instituto de Pesquisas de Produtos Naturais Walter Mors, Centro de Ciências da Saúde, Bloco H, Universidade Federal do Rio de Janeiro, 21941-902 Rio de Janeiro, RJ, Brazil

Correspondence should be addressed to José C. F. Alves; alvesjcf@yahoo.com.br

Table of Contents

Title of Manuscript and Author..1

Table of Contents……………………………………………………………………...2

1. Proposal of Synthesis of (+/-)-Goniomitine (**1**)…………………………………..…...3

1.1. Proposal of Synthesis of the Cyclopentanone **8**…………………………………..…3

1.2. Proposal of Synthesis of the Bromolactam **16**………………………………………4

1.3. Proposal of Final Steps for the Synthesis of (+/-)-Goniomitine (**1**)..………………..…5

1.4. Estimation of Overall Yield for the Synthesis of (+/-)-Goniomitine (**1**)…..………..5

2. Proposal of Synthesis of the Natural (-)-Goniomitine (**1**)…..………………………...6

2.1. Proposal of Synthesis of the Cyclopentanone **26**……………………………….....7

2.2. Proposal of Synthesis of the Chiral Cyclopentanone **8**……………………………..7

2.3. Proposal of Synthesis of the Chiral Bromolactam **16**……………………………..…8

2.4. Proposal of Final Steps for the Synthesis of (-)-Goniomitine (**1**)…..………………..…8

2.5. Estimation of Overall Yield for the Synthesis of (-)-Goniomitine (**1**)..………….…9

3. Proposal of Synthesis of the Unnatural (+)-Goniomitine (*ent*-**1**)..……………………9

References…………………………………………………………………………...10

This supplementary material displays the proposals of synthesis of the indole alkaloids (+/-)-goniomitine, the natural (-)-goniomitine, and the unnatural (+)-goniomitine. The idealized strategies and synthetic routes for the preparation of these alkaloids and stereisomers are described.

*1. Proposal of Synthesis of (+/-)-Goniomitine (**1**).* The convergent synthesis of (+/-)-goniomitine (**1**) was idealized by the retrosynthetic analysis depicted in Scheme S1.

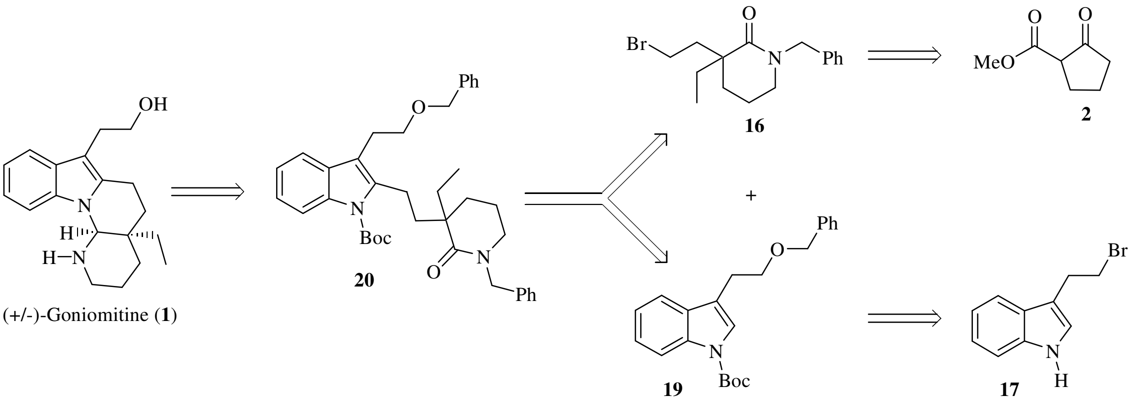

Scheme S1. Proposal of retrosynthetic analysis for the convergent synthesis of (+/-)-goniomitine (**1**).

The synthetic routes and conditions presented herein are based on previous experience of the author of this supplementary material on the synthesis of indole alkaloids [1-3].

The sequences of reactions proposed for the total synthesis of (+/-)-goniomitine (**1**) are described in the following subitems.

*1.1. Proposal of Synthesis of the Cyclopentanone **8***

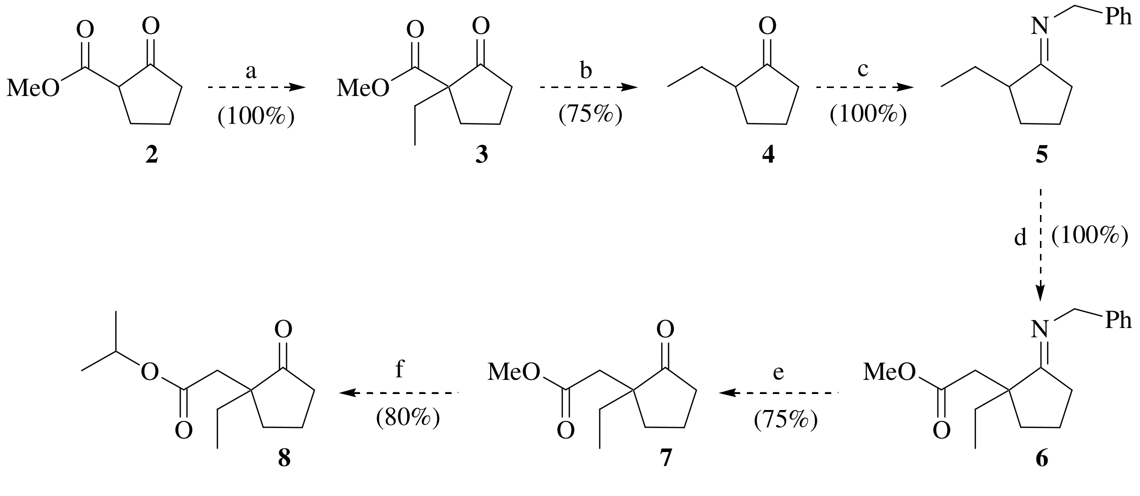

Scheme S2. Simulated conditions: (a) (i) Na_2CO_3 (4.0 equiv), acetone (1 mol L^{-1}), r.t. (30 min), (ii) CH_3CH_2I (2.0 equiv), reflux (6 h); (b) conc. HCl (2 mol L^{-1}), H_2O (10 mol L^{-1}), reflux (3.5 h); (c) Benzylamine (1.1 equiv), TsOH (0.02 equiv), toluene (0.5 mol L^{-1}), reflux (Dean-Stark, 5 h); (d) $BrCH_2CO_2Me$ (1.1 equiv), *t*-BuOH (3.3 mol L^{-1}), r.t. (24 h); (e) 10% AcOH-H_2O, THF (0.6 mol L^{-1}), r.t. (24 h); (f) *i*-PrONa (1.1 equiv), *i*-PrOH (0.3 mol L^{-1}), r.t. (15 min).

*1.2. Proposal of Synthesis of the Bromolactam **16***

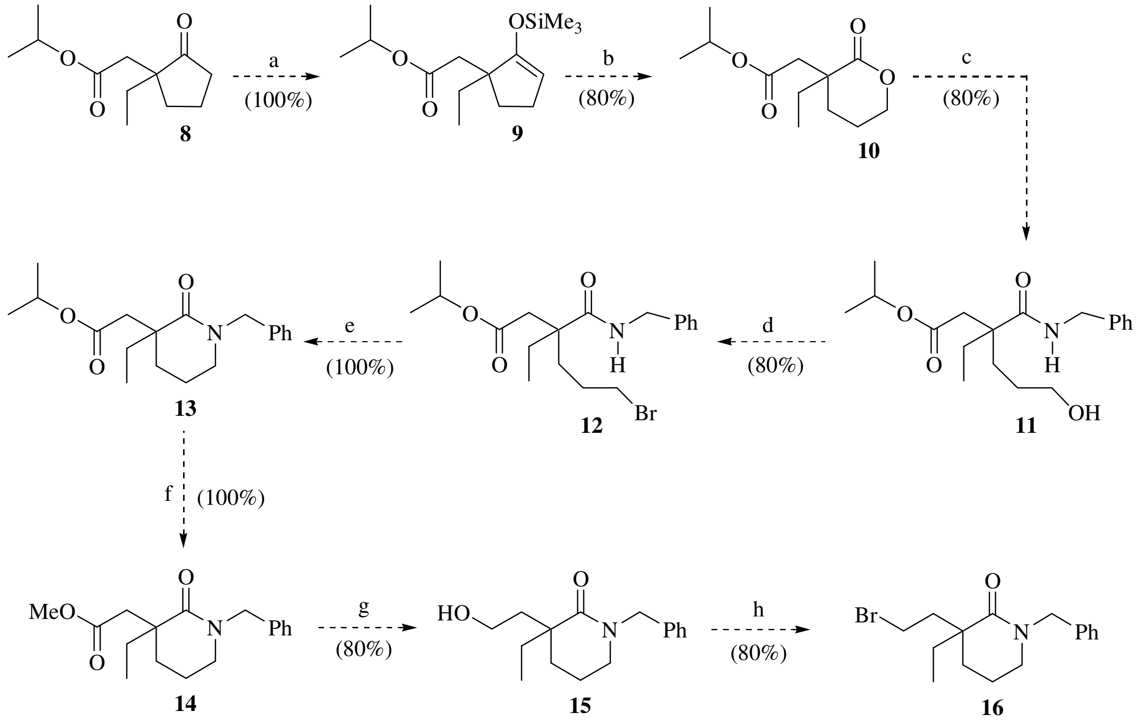

Scheme S3. Simulated conditions: (a) NaI (2.0 equiv), TMSCl (1.3 equiv), Et_3N (2.0 equiv), MeCN (1.13 mol L^{-1}), r.t. (30 min); (b) (i) O_3, 15% MeOH-CH_2Cl_2 (-78°C, 30 min), (ii) $NaBH_4$ (2.0 equiv), r.t. (3 h), (iii) 10% HCl-H_2O (pH 3), (iv) conc. HCl (cat.), CH_2Cl_2 (r.t., 3 h); (c) Benzylamine (1.2 equiv), CH_2Cl_2 (1 mol L^{-1}), 135°C (3 h); (d) CBr_4 (3.0 equiv), Ph_3P (1.5 equiv), CH_2Cl_2 (0.06 mol L^{-1}), r.t. (2 h); (e) KH (1.5 equiv), 18-crown-6 (0.6 equiv), THF (0.01 mol L^{-1}), r.t. (10 min); (f) MeONa (1.1 equiv), MeOH (0.02 mol L^{-1}), r.t. (1 h); (g) LiAl(*t*-BuO)$_3$H (2.0 equiv), THF (0.1 mol L^{-1}), r.t. (4 h); (h) CBr_4 (3.0 equiv), Ph_3P (1.5 equiv), CH_2Cl_2 (0.06 mol L^{-1}), r.t. (2 h).

*1.3. Proposal of Final Steps for the Synthesis of (+/-)-Goniomitine (**1**)*

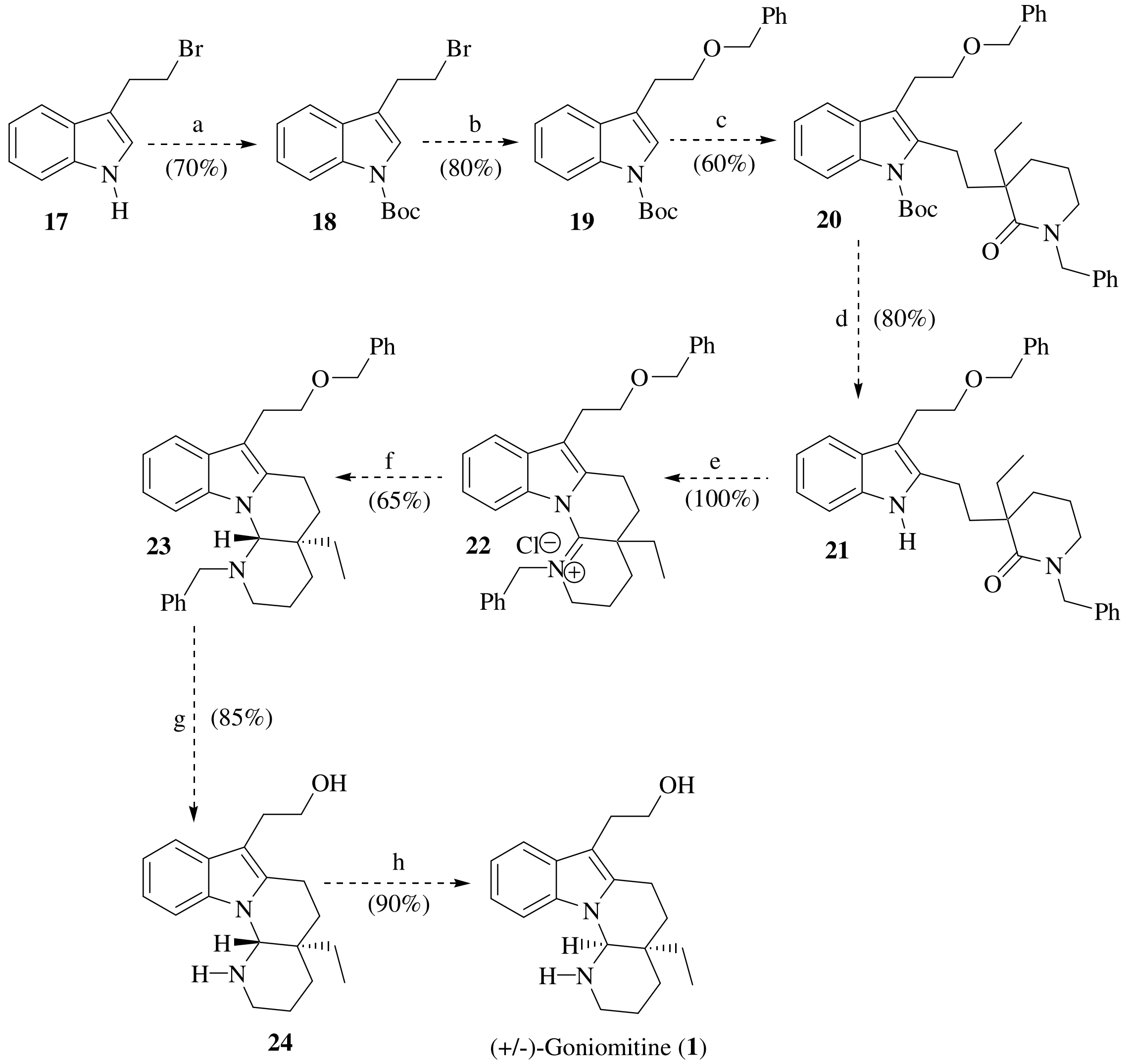

Scheme S4. Simulated conditions: (a) (i) NaH (1.2 equiv), THF (0.5 mol L^{-1}), 0°C (15 min), (ii) $(Boc)_2O$ (1.2 equiv), 0°C (30 min); (b) $C_6H_5CH_2ONa$ (1.2 equiv), MeCN (0.21 mol L^{-1}), 0°C (1 h); (c) (i) *n*-BuLi (1.1 equiv), -78°C (30 min), (ii) Compound **16** (1.0 equiv), THF (0.1 mol L^{-1}), [-78°C (30 min), r.t. (1 h)]; (d) TFA (1.1 equiv), CH_2Cl_2 (0.1 mol L^{-1}), r.t. (1 h); (e) $POCl_3$ (0.1 mol L^{-1}), reflux (5 h); (f) LiAl(*t*-BuO)$_3$H (1.3 equiv), THF (0.1 mol L^{-1}), 0°C (4 h); (g) H_2 (50 psi), 20% $Pd(OH)_2$ (0.1 equiv), 40% EtOH-AcOH (0.1 mol L^{-1}), r.t. (3 h); (h) 10% HCl-MeOH (0.08 mol L^{-1}) reflux (30 min).

*1.4. Estimation of Overall Yield for the Synthesis of (+/-)-Goniomitine (**1**).* Using the supposed yields presented in the simulated routes for the synthesis of (+/-)-goniomitine (**1**) (Schemes S2-S4), this target alkaloid may be obtained in 22 steps with 2% overall yield. Thus, using as starting material 2-carboxymethyl-cyclopentanone (**2**) (10 g), it is expected to obtain (+/-)-goniomitine (**1**) (0.42 g) (Scheme S5).

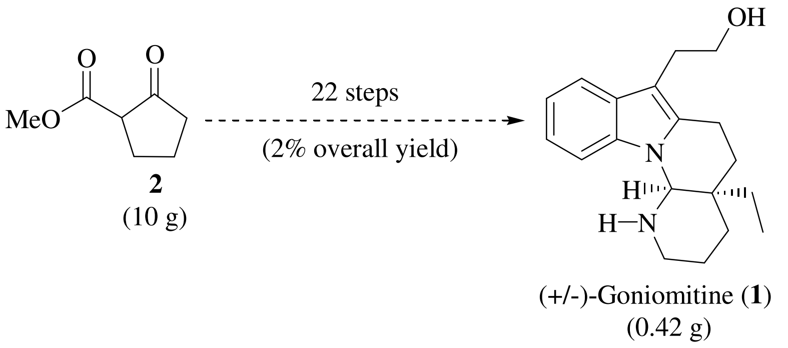

Scheme S5. Estimated overall yield for the synthesis of (+/-)-goniomitine (**1**).

2. Proposal of Synthesis of the Natural (-)-Goniomitine (1). The convergent synthesis of (-)-goniomitine (**1**) was idealized by the retrosynthetic analysis depicted in Scheme S6.

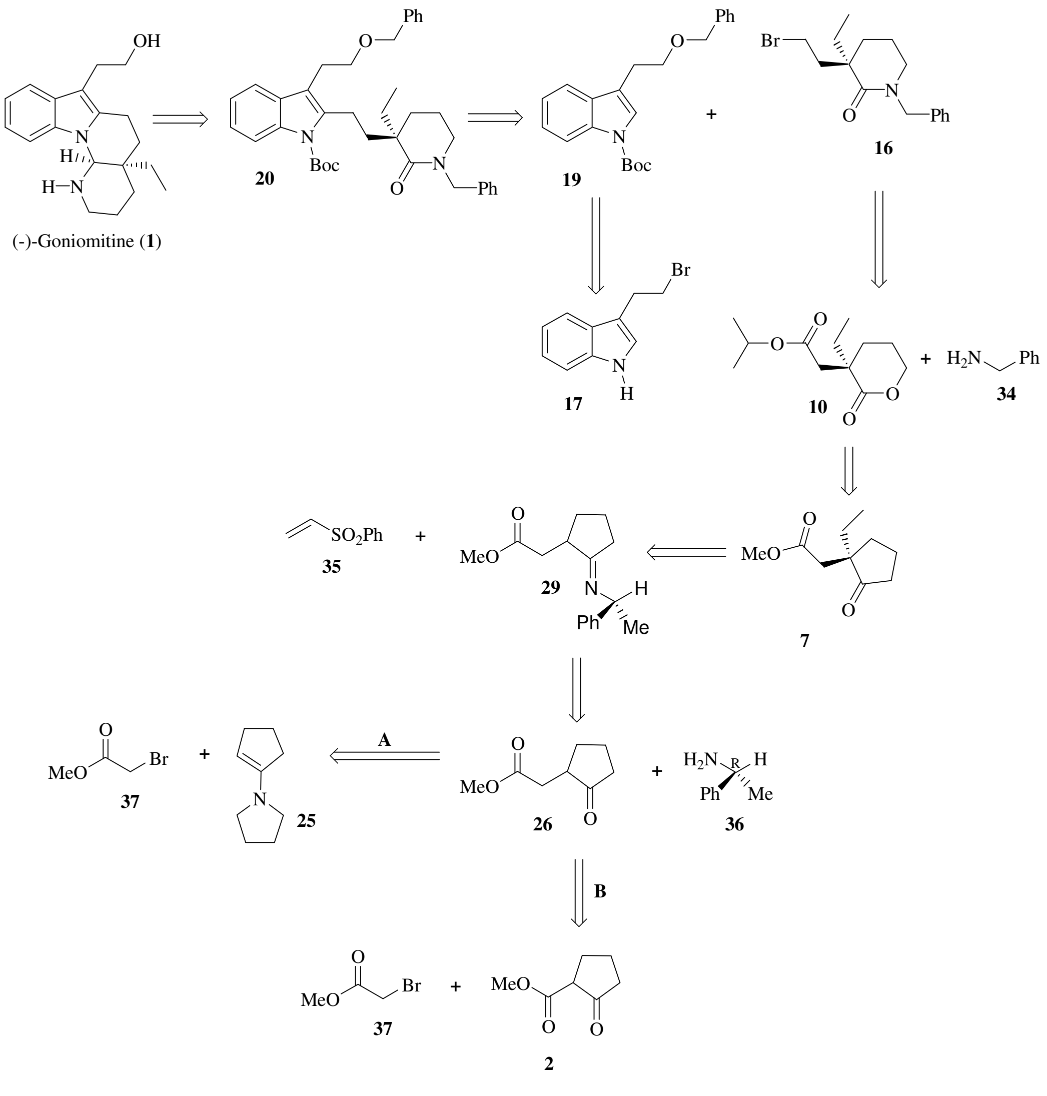

Scheme S6. Proposal of retrosynthetic analysis for the convergent synthesis of (-)-goniomitine (**1**).

The sequences of reactions proposed for the total synthesis of (-)-goniomitine (**1**) are described in the following subitems.

*2.1. Proposal of Synthesis of the Cyclopentanone **26***

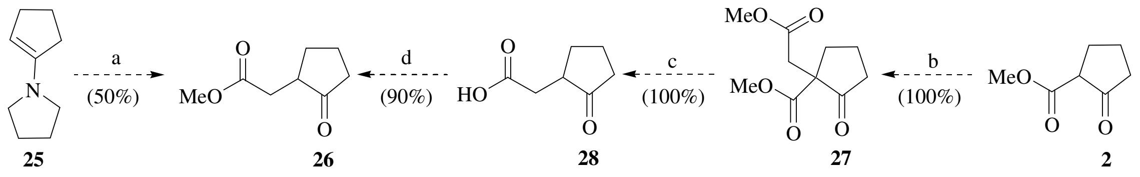

Scheme S7. Simulated conditions: *Procedure 1.* (a) (i) $BrCH_2CO_2Me$ (1.1 equiv), *t*-BuOH (3.3 mol L^{-1}), r.t. (12 h), (ii) 20% $AcOH-H_2O$, r.t. (4 h). *Procedure 2.* (b) (i) Na_2CO_3 (4.0 equiv), acetone (0.2 mol L^{-1}), r.t. (30 min), (ii) $BrCH_2CO_2Me$ (2.0 equiv), reflux (6 h); (c) conc. HCl (2 mol L^{-1}), H_2O (10 mol L^{-1}), reflux (3.5 h); (d) DCC (1.1 equiv), DMAP (0.1 equiv), MeOH (5.5 equiv), CH_2Cl_2 (0.08 mol L^{-1}), r.t. (15 h).

*2.2. Proposal of Synthesis of the Chiral Cyclopentanone **8***

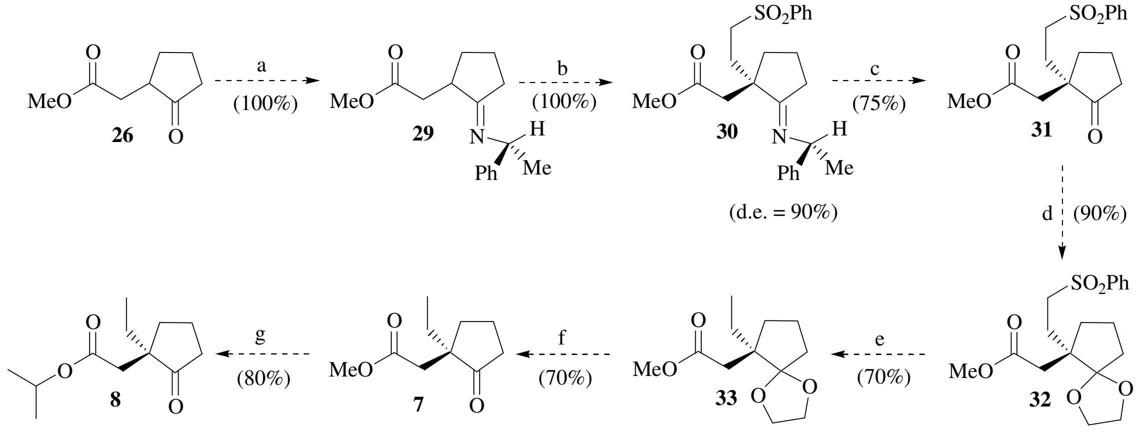

Scheme S8. Simulated conditions: (a) *R*-(+)-α-methylbenzylamine (1.1 equiv), toluene (0.37 mol L^{-1}), reflux (Dean-Stark, 24 h); (b) Phenyl vinyl sulfone (1.1 equiv), toluene (1.6 mol L^{-1}), 80°C (24 h); (c) 10% $AcOH-H_2O$, THF (0.6 mol L^{-1}), r.t. (36 h); (d) $HOCH_2CH_2OH$ (2.0 equiv), TsOH (0.4 equiv), toluene (0.26 mol L^{-1}), reflux (Dean-Stark, 24 h); (e) 6% Na(Hg), MeOH (0.1 mol L^{-1}), r.t. (1 h); (f) conc. HCl (cat.), THF (0.2 mol L^{-1}), r.t. (1.5 h); (g) *i*-PrONa (1.1 equiv), *i*-PrOH (0.3 mol L^{-1}), r.t. (15 min).

*2.3. Proposal of Synthesis of the Chiral Bromolactam **16***

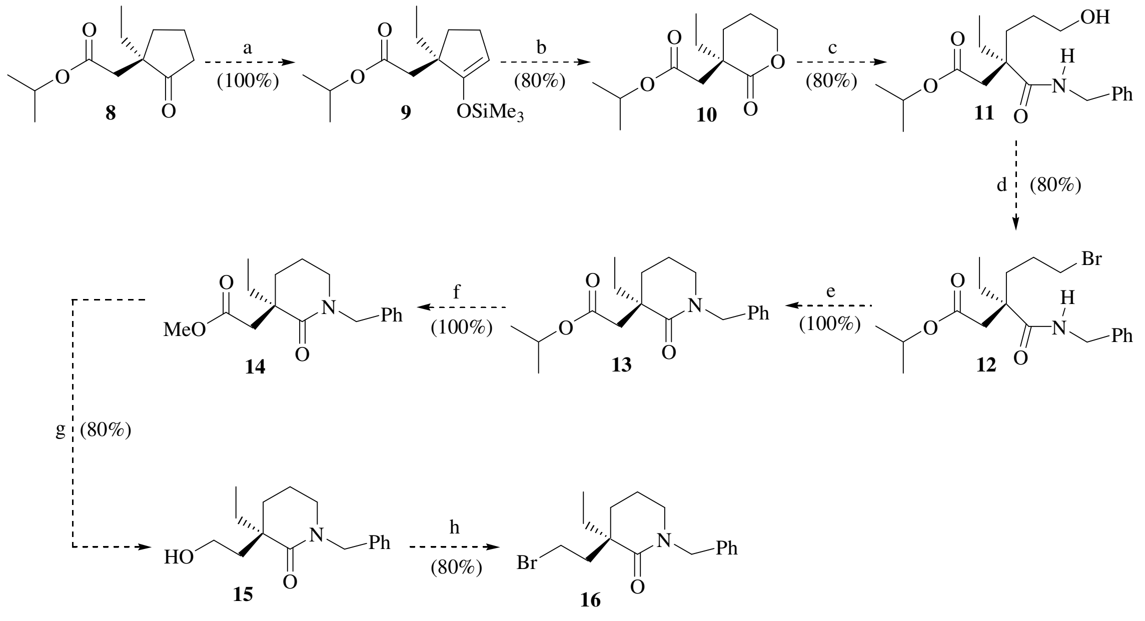

Scheme S9. Simulated conditions: see Scheme S3.

*2.4. Proposal of Final Steps for the Synthesis of (-)-Goniomitine (**1**)*

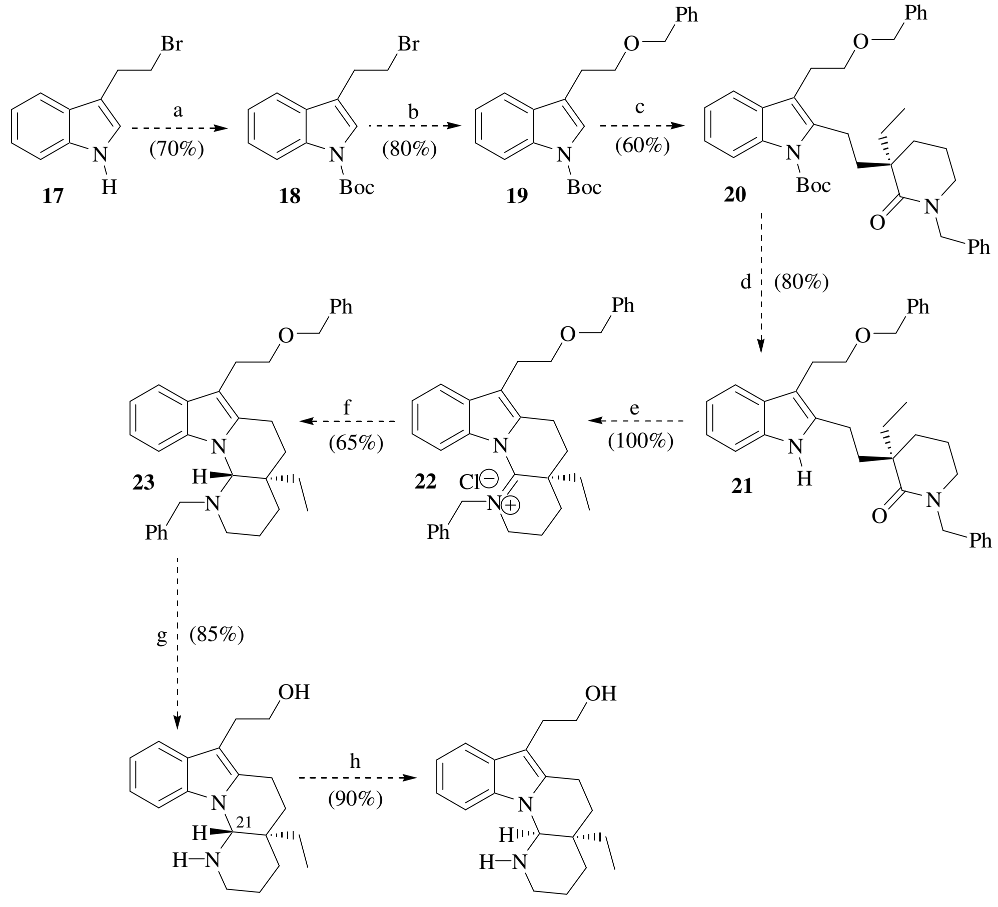

(+)-21-*epi*-Goniomitine (**24**) (-)-Goniomitine (**1**)

Scheme S10. Simulated conditions: see Scheme S4.

*2.5. Estimation of Overall Yield for the Synthesis of (-)-Goniomitine (**1**).* Using the supposed yields presented in the simulated routes for the synthesis of (-)-goniomitine (**1**) (Schemes S7-S10), this target alkaloid may be obtained in 24 steps with 0.6% overall yield (strategy **A**, Scheme S11), or it may be obtained in 26 steps with 1% overall yield (strategy **B**, Scheme S12).

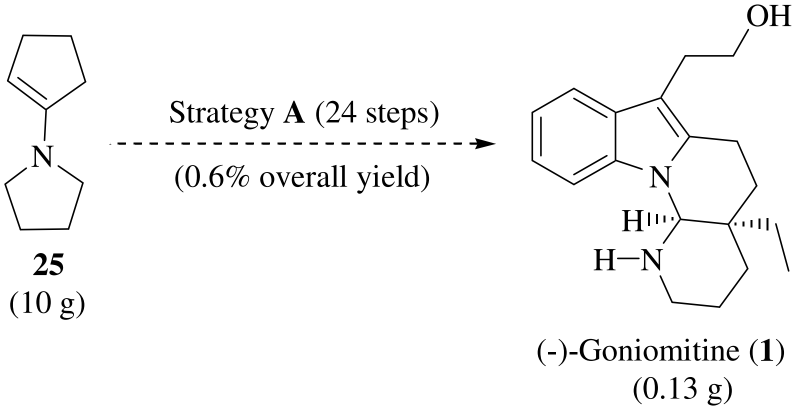

Scheme S11. Estimated overall yield for the synthesis of (-)-goniomitine (**1**), starting from 1-pyrrolidino-1-cyclopentene (**25**).

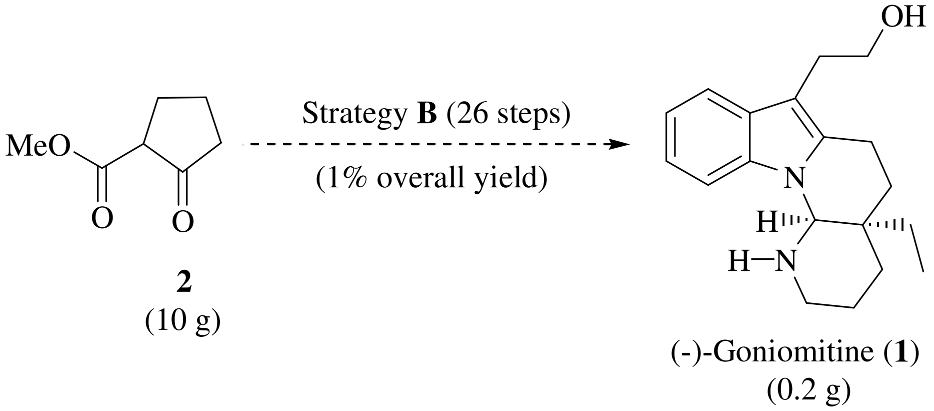

Scheme S12. Estimated overall yield for the synthesis of (-)-goniomitine (**1**), starting from 2-carboxymethyl-cyclopentanone (**2**).

*3. Proposal of Synthesis of the Unnatural (+)-Goniomitine (ent-**1**).* The synthesis of (+)-goniomitine (*ent*-**1**) was idealized starting from the enantiomer of the chiral imine **29** (*ent*-**29**), which may be synthesized in the reaction of the cyclopentanone **26** with the chiral amine *S*-(+)-α-methylbenzylamine (*ent*-**36**), using the same simulated conditions described for the synthesis of (-)-goniomitine (**1**) (see Scheme S13).

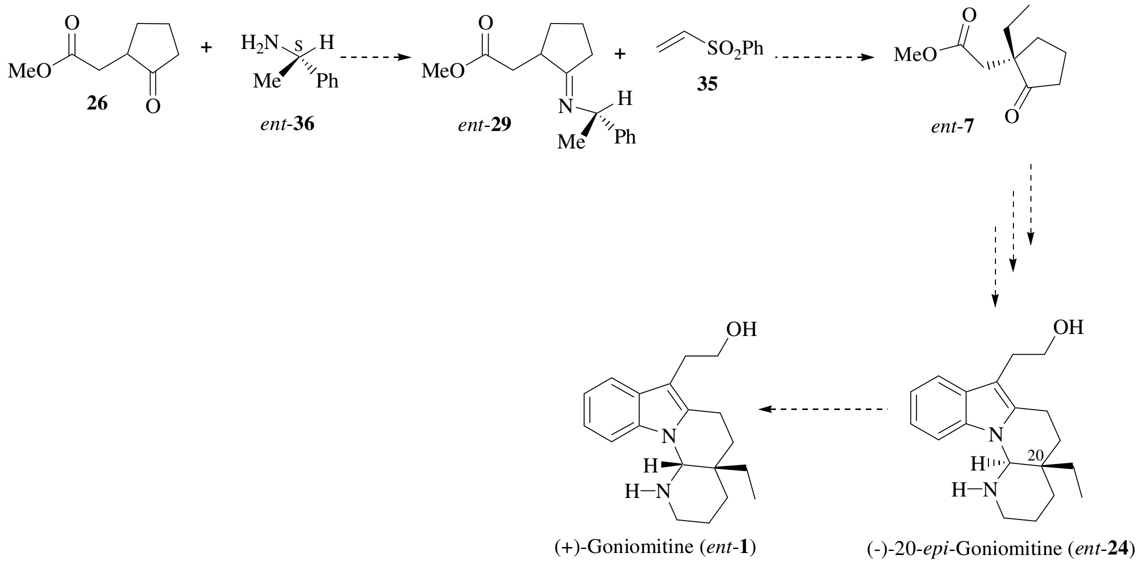

Scheme S13. Synthetic route proposed for the synthesis of (+)-goniomitine (*ent*-**1**).

References

[1] J. C. F. Alves, A. B. C. Simas, P. R. R. Costa, and J. d'Angelo, "Stereocontrolled elaboration of quaternary carbon centers involving the asymmetric Michael-type alkylation of chiral imines: an efficient enantioselective access to (+)-vincamine," *Tetrahedron: Asymmetry*, vol. 8, no. 12, pp. 1963-1966, 1997.

[2] J. C. F. Alves, A. B. C. Simas, and P. R. R. Costa, "Formal enantioselective synthesis of (+)-vincamine. The first enantioselective route to (+)-3,14-epivincamine and its enantiomer," *Tetrahedron: Asymmetry*, vol. 10, no. 2, pp. 297-306, 1999.

[3] J. C. F. Alves, *Formal enantioselective synthesis of (+)-vincamine and derivatives*, Ph.D. thesis, NPPN, Universidade Federal do Rio de Janeiro, Rio de Janeiro, Brazil, 2000.
